# Genetic Diversity of *ctxB* Gene Among Classical O1 and El Tor Strains of *Vibrio cholerae* using High-Resolution Melting Curve Analysis

**DOI:** 10.30699/ijp.2020.127793.2393

**Published:** 2020-07-15

**Authors:** Mahdieh Mahboobi, Reza Mirnejad, Hamid Sedighian, Vahhab Piranfar, Abbas Ali Imani Fooladi

**Affiliations:** 1 *Applied Microbiology Research Center, Systems Biology and Poisonings Institute, Baqiyatallah University of Medical Sciences, Tehran, Iran*; 2 *Molecular Biology Research Center Systems Biology and Poisonings Institute, Baqiyatallah University of Medical Sciences, Tehran, Iran*; 3 *Research and Development Department, Farname Inc, Thornhill, Canada*

**Keywords:** ctxB, Vibrio cholerae, High-resolution melt analysis, Genotyping Techniques

## Abstract

**Background & Objective:**

*Vibrio cholerae* is a natural inhabitant of the environment and causes severe diarrhea ailments (cholera) that affects thousands of people each year worldwide. The most important virulence factors of this pathogen are cholera toxin (cholera toxin CT) and Type IV pili (toxin co-regulated pili TCP), which are encoded within the genome of the filamentous bacteriophage CTXφ. In the present study, according to researchers’ report on genotypic variations of cholera toxin, we evaluated the sequence of *ctxB* subunit obtained from 100 strains of patients infected with cholera in Iran.

**Methods:**

The evaluation of genotype variations of cholera toxin was made by high-resolution melting curve analysis illustrating a single nucleotide change. Then, *ctxB* gene sequencing was performed. Through this analysis and the sequencing process, two standard samples were studied.

**Results:**

Using serologic tests, all the strains analyzed in this study were identified to be in O1 serotype. However, there have been differences in sequences of *ctxB* as some were similar to *V. cholerae* O1 biovar El Tor str. N16961 while others were similar to the genotype of *V. cholerae* ATCC 14035. We did not observe any particular pattern within the process of mutation.

**Conclusion:**

The analysis of the new samples of *ctxB *showed that they were potentially different. It seems that these complicated species were affected by a new genetic exchange of El Tor and classic genotypes.

## Introduction

*Vibrio cholerae* is a natural inhabitant of the environment and causes severe diarrhea ailments (cholera) that affects about three thousands of people and about one hundred deaths annually around the world (1). These gram-negative bacteria include more than 200 somatic O antigen serogroups (2,3). All of these serogroups cannot cause cholera and among them, serogroup O1 and O139 have caused several pandemics and epidemics. Serogroup O1 can be classified into two biotypes, classical and El Tor. Classical biotype is responsible for the sixth pandemic while biotype El Tor has caused the seventh pandemic in the past decade. In 1992, the serogroup O139 emerged from India and affected The Middle East and Asian countries as well (4-6).

The most important virulence factors of this pathogen are cholera toxin (CT) and toxin-coregulated pilus (TCP). TCP is important for the colonization of the bacteria in the host. The cholera toxin is encoded by *ctxA/B *operon that is a part of CTXφ genome (7). The *ctxA* is a reserved piece, which constructs the A subunit. The *ctxB* that is responsible for the production of the B subunit, plays the role of a receptor binding site of CT and varies among different genotypes (8-11).

Changes in *ctxB* gene led to the emergence of three classic genotypes in the Gulf of America, El Tor serotype in Australia and the former within the last pandemic of cholera was El Tor biotype strains. Three new genotypes 4, 5 and 6 of serotype O139 have been found in Bangladesh outbreaks (12-14). Genotype 5 resembles the classic genotype 1 in the Gulf of America. Similarly, genotype 6, with little changes in the amino acids, is similar to genotype 4. Previous reports showed that a new genotype existed in the serotype O3, which was responsible for further outbreaks. It seems that the outbreak of epidemics occurred in Asia, East India and some provinces of Iran is related to a new type of cholera toxin production similar to type 1 genotype (15-17).

In the early phase of the seventh pandemic of cholera, El Tor biovar was isolated. Furthermore, more unusual forms of El Tor were isolated either. These forms were observed in 1994 in Bangladesh. Moreover, in 2011, researchers discovered a few unusual biotypes of El Tor in Asia, America and Africa (2, 4). These unusual forms were El Tor varieties, which had the characteristics of the classical biotype. These studies reported that the biotype El Tor *ctxB* genes of *V. cholerae* were changing into the classical biotype (18). The occurrence of some phenotypic characteristics of classical biotype in El Tor biovar such as erythrocyte agglutination, Voges-Proskauer test and phage sensitivity assay confirm these changes (5). 

High-resolution melting curve (HRM) analysis is a simple, fast and useful method for mutation scanning of disease and genotyping of the viruses and bacteria (19). This method consists two simple steps, Polymerase Chain Reaction (PCR) and short melting. The melting step based on GC content, length, heterozygosity, and mutations in the sequence of the PCR product that make differences in the shape of the melting curve rather than wild type curve (20, 21). 

In this study, we evaluated the sequence analysis of *ctxB* subunit obtained from 100 samples of patients infected with cholera in Iran. Furthermore, the evaluation was made by high-resolution melting curve analysis illustrating a single nucleotide change. The analysis of the new samples of *ctxB* showed that they were potentially different. It seems that these complicated species were affected by a new genetic exchange of El Tor and classic genotypes.

##  Materials and Methods

**Bacterial Isolates**

A total of 100 strains of *V. cholerae* were isolated from patients infected with cholera over seven years (2010 to 2017) in Iran. Two species of *V. cholerae* Serotype Ogawa serovar O:1 ( ATCC 14035) and *Vibrio cholerae* O1 biovar El Tor str. N16961, as positive and standards samples were provided from the microbial bank of Shahid Beheshti University of Medical Sciences (Tehran, Iran). 

**Initial Isolation, Phenotypic and Serological Tests**

Standard clinical and environmental colonies of *V. cholerae* collected in alkaline peptone water were enriched and incubated in TCBS medium (HiMedia Laboratories, India) at 37°C for 24 hours based on the standard microbial culture procedure. After incubation, to the growth of yellow colonies, Gram staining, biochemical and serological tests were performed. The colonies grown from clinical isolates were cultured on a fresh TSI medium (HiMedia Laboratories, India) (2, 22). 

Serological test was conducted based on slide agglutination method with polyvalent O1 and O139 Inaba and ogawa antiserums, which were purchased from The Mast Diagnostics UK Company. This method is based on detection of agglutination as a result of reactions between bacterial surface antigens and added antiserum. Finally, all strains were suspended in BHI broth (Merck, Germany) supplemented with 50% glycerol and stored at -70°C.

**Genomic DNA Extraction**

*V. cholerae* DNA was extracted by the boiling method according to a standard procedure and was dissolved in DDW. The obtained products were Storage at -20 °C until the analysis. 

**Primer and Probe**

The oligonucleotide primers for *ctx*B with forward sequence 5'-TGAT TTGTGTGCAGAATACC -3' and reverse sequence 5'-GGGTATCCTTCATCCTTTC-3' with PCR product size of 193bp were designed based on existing GenBank sequences for all strains of *V. cholerae* by primer3 software. 

Furthermore, due to the use of TaqMan techniques for Real-Time PCR, a probe marked with TET reporter yellow color and TAMRA quencher with 5'-TET-ACCAGGTAGTCAACATATAGATTCACA-TAMRA-3 sequence was designed, as well.

**The** ***ctx*** **B Gene PCR and Optimization**

To confirm the isolated strains and the presence of the *ctx*B genes using PCR techniques. The PCR optimization for determination of optimal concentration of each component of the reaction mixture was performed by the methods previously described (23). For efficient amplification, the optimization of the MgCl2 concentration, the amount of DNA and the annealing temperature of the primers were determined. The optimized values of 1 μL (100 ng) DNA template, 1 μl of forward primer (10 pmol), and 1 μL of reverse primer (10 pmol) with 7 µL of deionized water were added to 12.5 µl of red ampliqon master mix (2X), purchased from Solis BioDyne, a Danish company (Total volume = 25 µL). The PCR cycles were ran under these conditions: initial denaturation at 95°C for 5 minutes, 35 cycles of denaturation followed by 95°C for 1 min, annealing at 53°C for 1 min, extension at 72°C for 30 Sec and the final extension at 72°C for 4 min. Eventually electrophoresis was performed on a 1.5% agarose gel at the voltage of 80 for 40 minutes (24).

**Real-Time PCR for** ***ctx*** **B Gene**

Real-Time PCR method was performed using ABI7500 device from an America Applied Bio-systems company with absolute quantization technique. For this purpose, the optimized concentrations contained 12.5 µL of 2x Real-Time probe master with ROX, 1 μL of forward primer (10 pmol), and 1 μL of reverse primer (10 pmol), 2 μl of Dual-labled DNA probe (10pM), 2.5 μL (100 ng) DNA template and 6 μL PCR-grade Water (final volume was 25 μL) were added to the device’s special micro-tube. The optimized temperature program was as follows. Initial denaturation at 95°C for 2 minutes, then 40 cycles of denaturation at 95°C for 15 seconds, annealing at 53°C for 30 seconds, extension at 72°C for 30 seconds and the final extension step was performed at 72°C for five minutes.

**High Resolution Analysis Curve**

The HRM method for clinical and standards samples was conducted with RotorGene 6000 device based on the manufacturer's instructions and the same scheduled melting temperature of 75°C by 0.1 degree steps up to 95°C. The 5x HOT FIREPol® EvaGreen® HRM Mix was used for this technique.

**Sequencing of the** ***ctx*** **B Fragment**

The *ctx*B gene fragment sequencing in clinical specimens was conducted and read for 3 times for each amplify fragments and the results were analyzed with Chromas software.

## Results

**Serologic and Phenotypic Tests**

A total of 100 clinical samples of *V. cholerae* were isolated by a standard culture on TCBS medium and serological test results confirmed the samples as an O1 serotype. 

Results of phenotypic tests for blood chicken agglutination (CCA) and Voges-Proskauer test were positive. In El Tor strains antibiotic resistance was observed to be 50 units of polymyxin.

**PCR and Real-Time PCR**

**Fig. 1:**
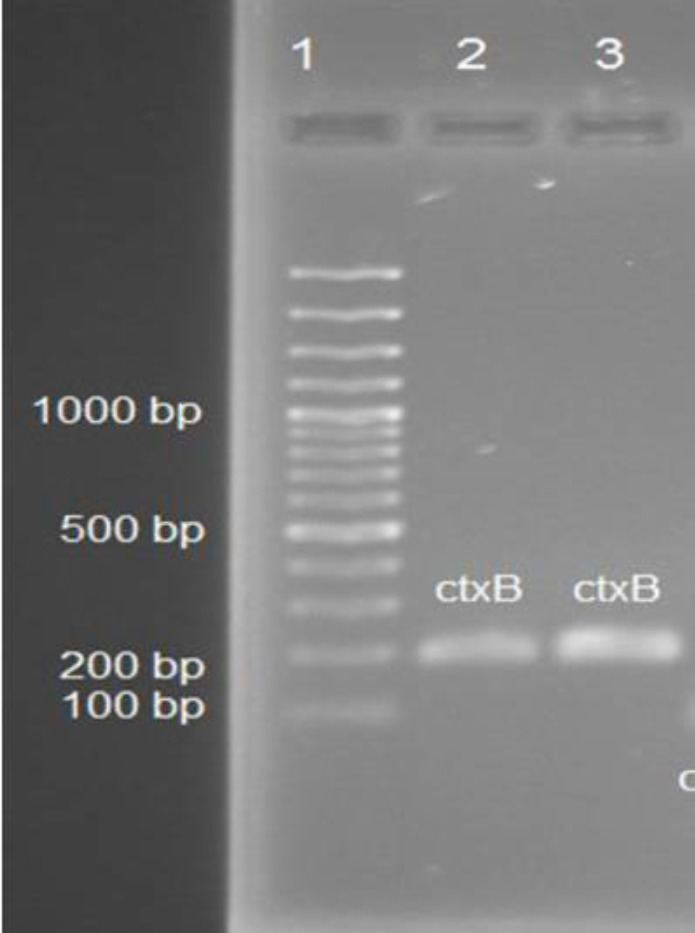
Gel analysis of the *ctx*b PCR product. The results of control and a clinical sample (as a model) on 1.5% agarose gel. Lane 1= DNA ladder, lane 2= The *ctx*B product of control strain and lane 3= The *ctx*B product of a clinical isolate

Genomic DNA of clinical samples was obtained using the boiling method (25). The quality of extracted DNA was better compared with that of Bioneer kit (Korea). Moreover, the presence of the *ctx*B gene in all samples was confirmed by PCR methods (Figure 1). Similarly, all samples were also identified and confirmed by Real time-PCR method

**Sequencing Results of** ***ctx*** **B Fragment**

**Fig. 2:**
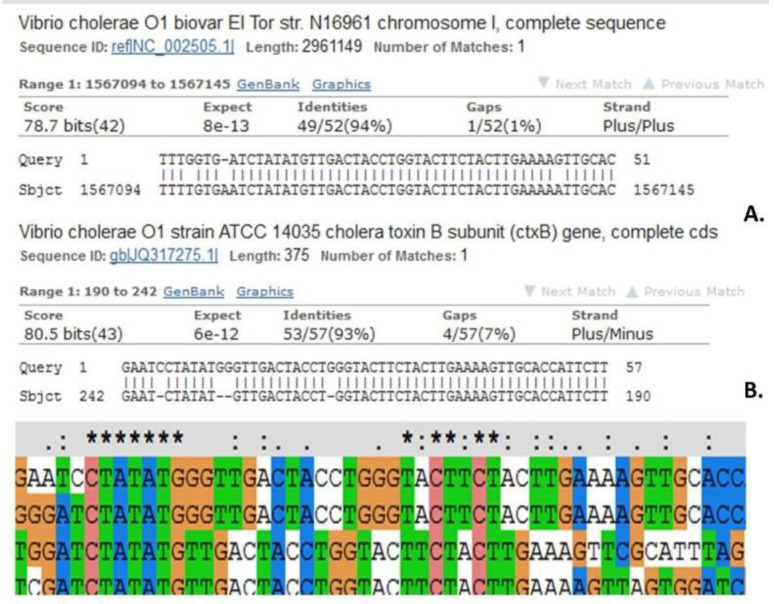
Sequence differentiation between the clinical samples and the standard samples**. **Indicates the sequencing results of amplified *ctx*B fragment in a clinical sample and the standard *V. cholerae* O1 biovar El Tor strain N16961 (figure a). Figure b indicates the results of amplified fragment *ctx*B in a clinical sample biovar El Tor, which shows a similarity to *ctx*B sequence of the standard *V. cholerae* ATCC 14035. The differences between standard samples and other clinical samples were identified using ClustalX 1.2 software. The results showed that *ctx*B fragment was conserved among clinical samples bearing mutations including deletions, transitions and insertions

Sequencing results of clinical samples revealed that all 100 samples were close to the standard strain of *V. cholerae* O1 biovar El Tor strain N16961. Figure 2 shows a clear difference between the clinical samples and the standard samples.

**HRM Results of** ***ctx*** **B Gene**

**Fig. 3:**
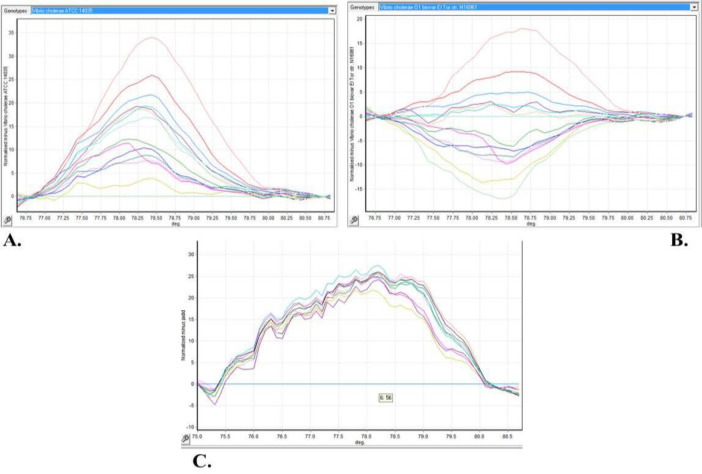
Melting curve analysis. The right and bottom-left diagrams show sorting samples according to genotype of *V. cholerae* O1 biovar El Tor strain N16961and *V. cholerae* ATCC 14035 respectively. Clinical samples from the top-left and bottom figures are different. Identified patterns show that the sequence of clinical samples varies from standard samples and included mutations. DNA concentration of all samples and extraction procedure method were the same

Melting curve analysis with high-quality results indicated that clinical samples have different types of mutations compared with the standard strains. The result of the experiment is depicted in Figure 3.

**Table 1:** *Optimized PCR reactions. The PCR parametrs for detection of CTXb *in *V. cholereae* clinical samples

Component	Volom for 1×25ul	Final conc.
2x Real-Time probe master (rox+)	12.5 µL	1×conc.
DNA TemplTE	2.5 µL	
Primer Forward (10 pM)	1 µL	400nM
Primer Reverse (10 pM)	1 µL	400nM
Dual-labled DNA probe(10pM)	2.0 µL	400nM
PCR-grade Water	6.0 µL	-

## Discussion

The *V. cholera* is a pathogen transmitted through contaminated water. The cholera toxin (CT) is the protein secreted during the infection. This toxin contains 1 A and 5 B subunits, which 5 B subunits play a role in attaching to the intestinal epithelial cells and contains different genotypes (26). In this study we examined the genotypes of *V. cholerae* and compared the samples with the existing standards. 

Samples were collected from epidemics pertaining to a variety of geological backgrounds in Iran. The noted samples were transferred to the research laboratory, the mutations in the B subunit of bacterial enterotoxins were examined using the new technique of HRM (27). For preserving the shape of the HRM diagram, samples, which show the CT value within 20 to 30. Furthermore, the results were compared with the sequencing. The study confirmed different mutations within the amplified fragment. 

The current literature lacks significant studies, which have been conducted to determine the differences in *ctx*B using HRM technique. Our study confirmed similar findings reported by other researchers in various countries regarding the genotypes of *V. cholera* illustrating that alteration in the phenotypic state can impose complex genotypic changes, which increases the genetic transfer (28,29). The detected changes are not that significant to be considered as a new species. Accordingly, samples were placed outside the El Tor group taxonomy.

Similar to our study, Le Roux* et al.*, analyzed the toxigenic and non-toxigenic *V. cholera* in environmental and clinical samples using the HRM technique to amplify *ctx*A/B and ompW fragments (30). The study concluded that the HRM technique is indeed a suitable method to differentiate between toxigenic and non-toxigenic clinical strains. As this technique does not require maintained probe and sequencing to hybridize the probe, it is considered a cheaper method compared with other techniques. In other studies, conducted by Jin* et al.*, and Piranfar* et al.*, the *Listeria* and Brucella species were identified and separated using HRM and EvaGreen color techniques, which facilitated the identification of samples (31-33).

All random samples used in this study were identified to be in O1 El Tor using serologic tests. However, there were differences in sequences of *ctx*B as some were similar to *V. cholerae* O1 biovar El Tor strain N16961 whereas some were similar to the genotype of *V. cholerae* ATCC 14035. We did not observe any particular pattern within the process of mutation; however, we observed that this fragment can create a complex composition under certain conditions, which can contribute to the production of a new genetic exchange from El Tor and classic genotypes. 

The latest findings confirm significant genetic changes in biotypes of *V. cholerae*. For instance, there is an El Tor *V. cholerae* biotype in Bangladesh, which produces the classic cholera toxin. Furthermore, investigations report a genetic shift in other countries such as Japan, which may present as an epidemic (9,30). 

Our findings confirmed that the noted genetic shifts occurred due to a complex error in replication and recombination in the *ctx*B gene. The previous epidemics such as the seventh *V. cholerae* pandemic were produced by normal genotypes of 2, 1 and 3 (2-4). The third El Tor biovar was produced in the above-mentioned pandemic and it was found in Australia. Newer genotypes, which were derived from genotype 3 were identified in Bangladesh a few years later (9, 13). 

The El Tor biovar is considered to have higher environmental resistance than the classic genotype while classic genotype tends to create evidently stronger symptoms (30). Consequently, the El Tor biobar with acquisition of the B subunit of classic genotype enterotoxin, which is responsible for the binding of the toxin, creates higher virulence and established environmental resistance (16). 

## Conclusion

Overall, our study substantiated that various sequences of *ctx*B in different strains of *V. cholerae* contain changes that facilitate the genetic exchanges, which can create a new type of *V. cholerae* bearing more potent virulence. Moreover, findings proved that HRM is a relatively sensitive and cheap hybridization technique, which lacks the need for consequent probe and sequence, while at the same time it can identify genetic modifications in sequences of *ctx*B toxigenic *V. cholerae*. Consequently, this technique may serve as a fitting tool for monitoring and differentiating toxigenic and non-toxigenic clinical strains from each other.
